# Seroprevalence of camel brucellosis in Qatar

**DOI:** 10.1007/s11250-022-03335-z

**Published:** 2022-10-20

**Authors:** Hashim Alhussain, Susu M. Zughaier, Ahmed Gawish, Mahmoud H. Mahmoud, Hadi M. Yassine, Asmaa Al Thani, Tahra El- Obied, Abdulaziz M. Al-Zeyara, Nahla O. Eltai

**Affiliations:** 1 Biomedical Research Center, Qatar University, P. O. Box 2713, Doha, Qatar; 2 College of Medicine, Qatar University, Health, Qatar University, P. O. Box 2713, Doha, Qatar; 3 Al Maha for Veterinary & Agriculture Services, Doha, Qatar; 4 Department of Animal Resources, Ministry of Municipality & Environment, Doha, Qatar; 5 Department of Human Nutrition, College of Health Sciences, Qatar University, Health, P. O. Box 2713, Doha, Qatar

**Keywords:** Brucellosis, Seroprevalence, Livestock, Camels, Qatar

## Abstract

Brucellosis is a significant zoonotic disease and one of the most common neglected diseases worldwide. It can infect a wide range of domestic and wild animal species. Infected animals are usually culled, causing substantial economic losses to animal owners and the country’s economy in general. The disease is endemic among cattle, sheep, and goats in many countries around the Middle East and prevalent in most Gulf Cooperation Council countries, comprising a significant public health risk in the region. This study investigated the seroprevalence of brucellosis among camels in Qatar. Two hundred and forty-eight samples were collected from dromedary camels from 28 farms across the entire country. Each sample was tested for *Brucella* antibodies with both Rose Bengal and competitive enzyme-linked immunosorbent assay. Only samples that tested positive by both tests were considered seropositive for brucellosis. The overall prevalence was (20.6%, 95% CI, 15.7–26.1). The association between sex and seropositivity was slightly significant (*Χ*^*2*^ = 4.32, *P* = 0.04), with higher seroprevalence in females. Camels below breeding age (i.e., < 4 years old) showed decreased seropositivity (3.4%, 95% CI, 0.1–17.8), compared to (22.8%, 95% CI, 17.4–29.0) seropositivity in camels ≥ 4 years of age, with a significant association between age groups and seropositivity (*P* = 0.02). Our results indicate that the seroprevalence of brucellosis in Qatar’s camels is alarming, mandating more efforts to control the disease. The findings of this study will aid in selecting better effective measures to control camel brucellosis in Qatar. Further studies need to be conducted on *Brucella* infection among camels to determine the predisposing risk factors and the steps that should be followed to control brucellosis.

## Introduction

*Brucella* species (spp.) is an intracellular facultative gram-negative coccobacillus non-motile bacterium (Wareth et al. 2017). The pathogen, renamed after its discoverer David Bruce (Moreno and Moriyón 2002), is the causative agent of brucellosis, one of the most common zoonosis worldwide (López-Santiago et al. 2019). The disease is variously known as undulant fever, Malta fever, and Mediterranean fever (Ahmadi et al. 2017). *Brucella* spp. can infect a wide range of domestic and wild animals, including sheep, goats, cattle, pigs, dogs, wild boar, hare, reindeer, caribou, rodent, ram, cetacean, seal, and fox (Godfroid et al. 2010).

Based on host preference and the phenotypes, *Brucella* genus is classically classified into six main species, *Brucella abortus* associated with cattle and camels, *Brucella melitensis* with sheep, goats, and camels, *Brucella canis* with dogs, *Brucella neotomae* with desert woodrats, *Brucella ovis* with sheep, and *Brucella suis* with pigs, reindeer, and hares (Rajendhran 2021). In animals, brucellosis causes abortion, premature births, metritis, reduced fertility, and reduced milk yield leading to significant financial losses (Franc et al. 2018). The disease is highly infectious in animals, and in the absence of suitable treatment, infected animals are usually culled, increasing the financial burden. Moreover, the identification of the disease in animals is challenging. The primary clinical signs (abortion and infertility) are neither pathognomonic nor unique and not manifested in all infected subjects (Ahmed et al. 2016).

Furthermore, animals can be latently infected with no clinical signs and symptoms and seronegative results making it harder to control or eradicate the disease from the herd or the country (Hou et al. 2019). Vaccines have played a crucial role in controlling bovine brucellosis for many years. However, due to the drawbacks of these vaccines, much effort has been undertaken to develop new vaccines that are safer, more effective, and can be used in other animals (Dorneles et al. 2015).

Brucellosis is still endemic in many developing countries (Mohamed et al. 2019), and several Gulf Cooperation Council (GCC) countries reported the disease among livestock animals (Radwan et al., 1992; Alrawahi et al., 2019; Mohamed et al., 2019; Al-Sherida et al. 2020). While Qatar has a low incidence rate of human brucellosis, compared to other countries in the region (Mohamed et al. 2019), the disease is more prevalent among livestock (Manivannan et al., 2021; MME, unpublished data). However, there is a lack of studies and scarce data regarding brucellosis among animals in Qatar.

Consequently, this study investigates brucellosis seroprevalence among camels in Qatar. Based on the latest statistics on the livestock population, Qatar had a census of 84,825 camels [MME, 2018 personal communication]. Camel products’ consumption and market share have grown in response to the increased demand and commercialization of camel products in Qatar, in addition, camel is an integral part of the local culture and heritage. Nowadays, a large percentage of camels in Qatar are racing camels which is a trendy sport in Qatar and the Arabian Gulf region; it is projected that Qatar had around 22,000 racing camels in 2016 (ELSA, 2016). Breeding camels are also very popular in the local market and among animal farm owners.

Moreover, camel meat and milk are also common in Qatar and across the Middle East and Northeast Africa. All this make it essential to analyze this disease’s status in camels and other local animals. Sera from non-vaccinated camels raised in different geographical regions in Qatar were screened for *Brucella* antibodies by Rose Bengal test (RBT) and competitive enzyme-linked immunosorbent assay (c-ELISA), to investigate the seroprevalence of the diseases in this subpopulation.

## Methods

### Study area design

An analysis of cross-sectional serological data was conducted in all eight municipalities in Qatar, namely, Doha, Shamal, Khor, Daayen, Umm-Salal, Rayyan, Shahaniya, and Wakrah. Stratified random sampling was applied in two stages, selecting herds randomly and then arbitrarily choosing animals within the herd.

Each municipality constituted a separate stratum. The number of camel population for each municipality was obtained from the Ministry of Municipality (MM).

### Total sample size

The minimum required sample size was calculated based on the equation $N=\frac{{4z}_\alpha^2p(1-p)}{W^2}={(\frac{z_\alpha}E)}^2$ p (1-p), where *N* is the sample size; *p* is the expected proportion who have brucellosis; *W* is the width of the confidence interval (equal to twice the margin of error); *E* is the margin of error (half the width, *W*) and Zα is a value from the normal distribution representing the confidence level (equal to 1.96 at 95% confidence level). Considering the estimated seroprevalence of 20% brucellosis among livestock, and if *W* = 0.1, our estimated minimum required size will be 246. The estimated seroprevalence was based on this research team’s pilot study on camels and sheep (unpublished data).

Two hundred and forty-eight blood samples were collected from 28 different farms. The number of samples collected from each municipality was proportional to the camel census in each municipality.

### Farm selection

Members of the research team identified the camel-housing farms in each municipality. Each farm was assigned an identification serial number; after that, SPSS statistics 24 (Statistical Package for the Social Science; SPSS Inc., Chicago, IL, USA) was used to randomly select from the serially numbered farms in each municipality.

### Animal selection

Each livestock animal in the state of Qatar, including camels, is tagged with a 15-digit animal identification number, which can be visualized using an ultraviolet light scanner. The animal identification number was used in the chosen farms to select camels randomly from the herd using SPSS.

### Sample size per municipality

The number of camel population in each municipality was obtained from the MM. The number of camels sampled from a given municipality was equivalent to the percentage of the total number of camels in that municipality to the total number of camels in Qatar. First, the percentage of camels in the municipality was calculated using the equation, $total\;number\;of\;camels\;in\;municipality\div\;total\;number\;of\;camels\;in\;Qater\;\times100$. Then, the *obtained percentile* was used to calculate the required number of samples as follows, $obtained\;percentile\;\div\;100\times sample\;size\;\left(248\right)$.

### Inclusion criteria

Camels of any age or sex that are apparently healthy with no history of brucellosis or vaccination were included in this study.

### Sample collection and preparation

The samples were collected over 1 year, from 30 November 2019 to 5 November 2020, by a licensed veterinarian. Approximately 5 ml of blood was collected from the jugular vein in serum separator tubes (BD SST™ II Advance). After collection, the samples were placed in cool boxes (4–8 °C). Then, directly transferred to the Microbiology Lab, Biomedical Research Center, Qatar University. Upon arrival, the serum was separated by centrifugation of the blood at 3000 g for 10 min, transferred to 1.5 ml Eppendorf tubes, and kept at − 20 °C for subsequent analysis.

A data collection sheet was used to collect information that included the animal identification number, sex, age, farm Identification number, and municipality during sampling.

### Seropositivity

Each sample was tested by the RBT (BENGATEST, SYNBIOTICS Europe-2, RUE Alexander Fleming-69367 LYON CEDEX 07—France) and c-ELISA (SVANOVIR®Brucella-Ab c-ELISA, Sweden). A sample was considered positive only when both tests showed positive results, i.e., samples positive by only one of the two tests were considered negative in the prevalence analysis.

### Rose Bengal test and c-ELISA

RBT was performed following the manufacturer’s instructions. Briefly, 30 µl of serum was added to an equal amount of BENGATEST solution in a glass slide at room temperature and mixed. The slide was then agitated for 4 min. The samples were considered Rose Bengal positive if any agglutination was observed.

#### C-ELISA

The procedure was performed following the SVANOVIR® kit manufacturer’s instructions. Five microliters of the sample/control was diluted with 45 µl of sample dilution buffer inside a well coated with *Brucella abortus* smooth lipopolysaccharide. After that, 50 µl of mouse antibody solution was added to the wells. The wells were then incubated for 30 min before washing and adding 100 µl of conjugate solution. After another 30-min incubation and a second washing step, 100 µl of substrate solution was added to the wells. Finally, the reaction was stopped by adding 50 µl of stop solution 10 min after adding the substrate solution. The optical densities were measured at 450 nm using a Multiskan™ FC microplate photometer machine (Thermo Scientific, USA). Lastly, the percent inhibition value was calculated to determine the positivity of the samples as follows, $100-(sample's\,optical\,density\times100\,\div\;Conjugate\,control's\,optical\,density)$. Samples with a 30% and above percent inhibition value were considered c-ELISA positive.

### Statistical analysis

All data collected from the field were entered in Microsoft Excel 2016 (Microsoft Corp., Washington, DC, USA). After checking the data integrity, it was transferred to SPSS Statistics 24. Descriptive analysis and Clopper-Pearson exact method were performed to determine the overall seroprevalence of camel brucellosis in Qatar and its municipalities. *χ*^2^ test was executed to identify the univariable association between brucellosis and different factors, including age and sex while regression models were used for the multivariate analysis. A probability value (*P*-value) less than 0.05 was considered statistically significant.

## Results

### Seropositivity

Of the 248 samples, 51 (20.6%, 95% CI 15.7–26.1) samples were positive (i.e., RBT and c-ELISA positive), and 197 (79.4%) were negative. Fifty-two samples (21%, 95% CI 16.1–26.6) were RBT-only positive, and 55 (22.2%, 95% CI 17.2–27.9) were c-ELISA positive. Cohen’s *κ* was run to determine the agreement between the two serological tests. There was almost perfect agreement between the two tests, *κ* = 0.94 (95% CI, 0.30–0.89), *P* < 0.001.

### Age and sex as risk factors

The average age was 5.52 years in males and 8.96 in females. First, a univariable analysis using the chi-squared test was used to assess each risk factor individually. Then, multivariable analysis was performed using logistic regression to encompass the confounding factors and the interactions between the variables. The camels were subgrouped based on age to camels of non-breeding and breeding age groups. The non-breeding age group included camels younger than 4 years of age, while the breeding age group included camels at 4 years of age and older (Yasin and Wahid 1957; J A Skidmore 2003). Only (3.4%, 95% CI, 0.1–17.8) of the non-breeding age group samples were positive compared to (22.8%, 95% CI, 17.4–29.0) in the breeding group. Univariable analysis showed a statistically significant association between age group and seropositivity (*P* = 0.02). Within the breeding age group, camels of age 4 to 6 years showed the highest percentage of seropositivity in which (27.8%, 95% CI, 14.2–45.2) of camels were positive compared to (21.9%, 95% CI, 16.1–28.6) seropositivity in camels older than 6 years of age; nevertheless, the difference was not statistically significant (Table 1).

**Table 1:** Univariable analysis of age and seropositivity association using Pearson’s chi-squared test

Age group (in years)	Number of the positive sample within the group and the total number of samples in the group	Percentage and 95% CI	*X*^*2*^ value	Pearson’s chi-squared *P*-value
Non-breeding against breeding group				
Younger than 4 (non-breeding)	1/29	3.4% (0.1–17.8)	5.89	0.02
From 4 to 15 (breeding)	50/219	22.8% (17.4–29.0)		
Within the breeding group				
Age 4 to 6	10/36	27.8% (14.2–45.2)	0.60	0.44
Age 7 to 15	40/183	21.9% (16.1–28.6)		

As for sex, of the 248 samples, 31 were males, and 217 were females. Female camels showed a higher percentage of seropositivity compared to males. Forty-nine (22.6%, 95% CI, 17.2–28.7) of females were seropositive, whereas two (6.5%, 95% CI, 0.8–21.4) of males showed positive results. Thus, the association between sex and seropositivity was slightly statistically significant (*Χ*^*2*^ = 4.32, *P* = 0.04).

However, the multivariable analysis, including both age and sex, revealed no statistical significance (Table 2).

**Table 2:** Multivariable analysis of the risk factors associated with brucellosis seropositivity

Variable	Category	Odds ratio	95% confidence interval	*P*-value
Age	Non-breeding	Ref	0.7–46.3	0.098
	Breeding	5.8		
Sex	Male	Ref	0.5–11.5	0.24
	Female	2.5		

### Municipality and farms

**Fig. 1:**
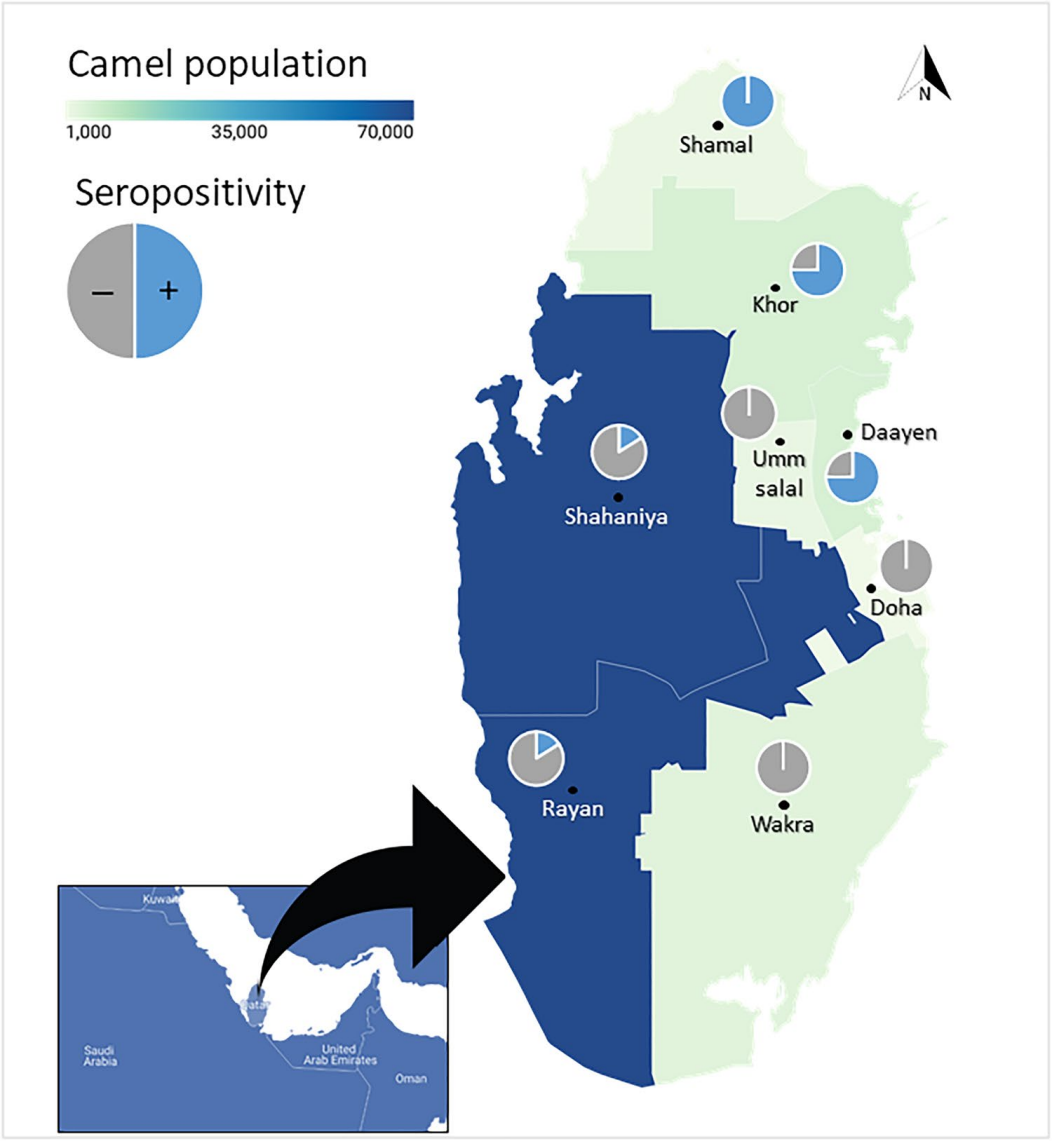
Distribution of camel population and seropositivity in the eight municipalities of Qatar

Samples were collected proportionally to the total number of camels in each municipality. The camel populations in Khor and Daayen and Rayan and Shahaniya were combined because few or no animals or farms were located in Daayan and Rayan. Rayyan-Shahaniya municipalities had more positive samples than any other municipality. Out of all the positive samples, 64.6% were from Rayyan-Shahaniya, followed by Khor-Daayen (23.5%), then Shamal (11.8%), while Wakra, Um-salal, and Doha had no positive samples (Table 3). The study surveyed 28 farms, of which 17 (60.7%) housed seropositive camels. Figure 1 shows the distribution of the camel population in the eight municipalities of Qatar and the seropositivity in each municipality.

**Table 3:** Camel population and percentage, number of samples, number and percentage of positive samples, number of selected farms, and number and percentage of positive farms in each municipality

Municipality	Camel number/percentage	Number of samples	Number, percentage, and 95% CI of positive samples	Number of selected farms	Number/percentage of positive farms
Khor-Daayen	5651 (6.7%)	16	12 (75%, 95% CI, 47.6–92.7)	2	2 (100%)
Rayan-Shahaniya	70,256 (82.8%)	205	33 (16.1%, 95% CI, 11.3–21.9)	21	14 (66.6%)
Shamal	1970 (2.3%)	6	6 (100%, 95% CI, 54.1–100)	1	1 (100%)
Wakra	3976 (4.7%)	12	0 (0%)	2	0 (0%)
Umm-salal	2326 (2.7%)	7	0 (0%)	1	0 (0%)
Doha	646 (0.8%)	2	0 (0%)	1	0 (0%)
**Total**	**84,825 (100%)**	**248**	**51 (20.6%, 95%CI, 15.7**–**26.1)**	**28**	**17 (60.7%)**

## Discussion

This is the first study investigating the seroprevalence of brucellosis among camels throughout the entire municipalities of Qatar. Laboratory diagnosis of brucellosis can be confirmed by a wide range of tests, including cultures (Hajia 2018), molecular (Çiftci et al. 2017; Batinga et al. 2018), and serological tests (Matope et al. 2011a, b). Each method has its limitations, and none is by itself sufficient to investigate brucellosis. Thus, it is necessary to perform at least two tests to confirm brucellosis (Michael J, 2006). In this study, RBT and c-ELISA were performed in parallel to increase certainty in estimating the disease prevalence rate.

In the present study, a total of 57 (22.9%) samples were positive by either of the two tests; from those, five were c-ELISA positive/RBT negative, and one was RBT positive/c-ELISA negative, while the total number of samples positive by both tests was 51 samples (Table 4). The seropositivity results were very similar in both tests, and Cohen’s *κ* revealed an almost perfect agreement between the two tests.

**Table 4:** Crosstab table showing the differences between RBT and c-ELISA results

	c-ELISA positive	c-ELISA negative
RBT positive	51	1
RBT negative	5	192

The relatively high seropositivity rates indicate the endemicity of brucellosis among camel populations in the country. The prevalence of brucellosis among camels was alarming and higher than that reported in other GCC studies. A study from Saudi Arabia in 1992 (Radwan et al. 1992) included 2630 blood samples and recorded an 8% seroprevalence. In Kuwait, Al-Khalaf and El-Khaladi reported a seroprevalence between 8.0 and 14.8% using different serological tests (Al-Khalaf and El-Khaladi 1989). Another study from Abu Dhabi, United Arab Emirates, investigated camel seroprevalence between 1991 and 1996 (Moustafa et al. 1998). They reported a decrease in seroprevalence over the years; it was 5.8% in 1991 and 0.1% by 1996. Due to the old date of these studies, they may not reflect the current status of camel brucellosis in these countries. A more recent study from Abu Dhabi reported a 4.4% camel brucellosis seroprevalence (Mohammed et al. 2013). Another recent and comprehensive study from Oman included 2250 samples from 552 herds/farms distributed across the country. Similar to the present study, the number of samples collected from each district was determined according to the camel’s population proportion. RBT and c-ELISA were used to confirm the positivity status of the sample. A very low seroprevalence was reported in the study with only nine samples (0.4%) from eight herds (1.5%) confirmed as seropositive (Alrawahi et al. 2019). All these studies surveyed a larger number of samples than the present study, which might explain the lower prevalence of the disease in those countries. However, it is more likely that the lower prevalence is a direct consequence of the vaccination or control programs implemented in these countries. Finally, Dadar et al. conducted a global meta-epidemiological study concerning the prevalence of camel brucellosis. In that study, the estimated seroprevalence (i.e., pooled prevalence) was 13.32%, 10.93%, 4.98%, and 0.34% in Saudi Arabia, Kuwait, United Arab Emirates, and Oman, respectively (Dadar et al. 2022). A disadvantage of that meta-epidemiological study was the inclusion of old studies in the analysis. All studies from 1980 to 2021 were included. From all the abovementioned studies and the present study, it appears that Qatar, Saudi Arabia, and Kuwait have relatively high seroprevalence compared to the United Arab Emirates and Oman.

Only one study was published from Qatar concerning brucellosis and camels; the study investigated immunological and molecular methods for the detection of brucellosis in camels (Manivannan et al. 2021). Although the author’s aim in that study was not to determine the prevalence of the disease in Qatar’s camel population, it provided evidence of high prevalence in Shahaniya municipality.

On average, camels mature and reproduce at age four, reaching full reproductive vigor by six. (Yasin and Wahid 1957; J A Skidmore 2003). This survey studied two main age categories of camels: camels younger than 4 years of age and camels aged four and older. This division between the age groups was necessary because the transmission of brucellosis is evidently most frequent in sexually mature animals (Hou et al. 2019). While young animals can get infected—usually without any clinical manifestations—it occurs with much lower prevalence (Hou et al. 2019). In the present study, camels younger than 4 years (non-breeding age group) had a much lower prevalence (3.4%, 95% CI, 0.1–17.8) when compared with camels over 4 years of age (breeding age group, 4–15 years) (22.8%, 95% CI, 17.4–29.0) (Table 1). The higher prevalence in mature camels is consistent with the findings of other studies investigating brucellosis in camels (Alrawahi et al. 2019) and other livestock animals, including buffaloes, cattle, sheep, and goats (Saeed et al. 2020). Several species of *Brucella* can grow in the reproductive tract due to erythritol production, a 4-carbon sugar produced in the fetal tissues of ruminants (Petersen et al. 2013). Thus, it may also explain the higher prevalence in adult camels than in young ones.

On the other hand, the higher prevalence of brucellosis in adults has also been linked to more prolonged contact with infected animals or the environment. This potential risk may be significant in herds where positive animals have not been culled. The association between age and seropositivity was further investigated by subdividing the breeding age group into two subgroups. The first subgroup included camels at the age of four to six, the age of sexual maturity up to the age of full reproductive vigor. The second subgroup included camels older than six; however, although the seroprevalence in the first group was higher (27.8%, 95% CI, 14.2–45.2) than in the second group (21.9%, 95% CI, 16.1–28.6), there was no statistically significant association between the two subgroups and seropositivity. Considering the debilitating nature of the disease, the lower seropositivity in the second subgroup (older than six) can be ascribed to the continues removal of elder infected animals from the herd as they become easier to detect with time due to their physical poor condition or repeated abortion over several years; however, this was not investigated in the current study.

The seroprevalence of camel brucellosis in females was higher than in males. Twenty-two percent of females were seropositive compared to 6.5% of males. Researchers had studied the influence of sex on the prevalence of brucellosis in cattle, small ruminants, and wild animals (Muma et al., 2006; Solorio-Rivera et al., 2007; Muñoz et al., 2010; C. Michael J, 2006). Several studies found a higher seroprevalence of brucellosis in females in camels (Alrawahi et al. 2019) and other animals (Teklue et al., 2013; Azami et al., 2018; Ndazigaruye et al., 2018; Saeed et al. 2020). Although difficult to explain, it may be related to the intrinsic biology of the *Brucella spp*. and its affinity for the fetal tissues, as previously described. In this study, the higher prevalence of seropositivity of brucellosis among females may also be attributed to a larger female sample size (*n* = 271) compared to the males (*n* = 31). Additionally, the age difference between the two sex groups might have also contributed as the average female age in the investigated sample was older (8.96 years) than that of males (5.52 years), and as aforementioned seropositivity tends to increase with the increase in age. Other studies however reported opposite results (Gabli et al. 2015) or no difference between sexes (Matope et al. 2011a, b).

The risk factor analysis was performed separately for age and sex using the chi-squared test and jointly using logistic regression. Univariable analysis showed a statistically significant association between seropositivity and age (*Χ*^*2*^ = 5.89, *P* = 0.02) and sex (*Χ*^*2*^ = 4.32, *P* = 0.04). However, the multivariable analysis showed no statistical significance in the association between age, sex, and seropositivity.

The Rayyan-Shahaniya municipalities accounted for more than 80% of all camel population in Qatar, while other camels are sparsely distributed in all other areas in the country. Nevertheless, due to limited sample size and financial constraints, additional studies involving a larger sample size and covering more farms should be conducted to determine the prevalence in specific municipalities. The tests were conducted on 22 samples from Khor-Daayen, and Shamal, with 81.8% being positive for *Brucella* antibodies. In Rayan-Shahaniya, from where most specimens were collected (*n* = 205/248), the prevalence was 16.1%. More than half (65.6%) of the positive samples were collected from Rayyan-Shahaniya, 23.5% were collected from Khor-Daayen, and the remaining 11.8% were from Shamal. Importantly, sixty percent of the farms included in the study housed seropositive animals; while it is unclear why so many farms housed infected animals when the total seroprevalence was comparatively low, it might indicate that the number of infected animals per farm is low, or at least that many farms housed a low number of infected animals. We think these results can be ascribed to the habit of exchanging camels between farm owners for breeding and other purposes. Nonetheless, the public health consequences of the presented results can be severe.

In conclusion, the seroprevalence of brucellosis in camels is alarming in Qatar. Further studies are required to understand the risk factors associated with disease transmission dynamics among the camel population. Consequently, there is a need to control the spread of the disease from camels to camels, other livestock, and humans. Different measures can be taken to reduce disease spread and exposure to public health, including using an effective and safe vaccine, following good biosecurity practices on farms, and increasing public awareness.

## Limitations

While the sample size is adequate to estimate the overall seroprevalence of camel brucellosis in Qatar, it might be considered inadequate to give concrete conclusions regarding the association between the disease and sex or age. In addition, we have chosen in this study to collect samples from each municipality in proportion to the camel population; while this approach has its advantages, it resulted in a significantly low number of samples in numerous municipalities; these municipalities might need to be investigated separately with a larger sample size to give a better idea about the seroprevalence in them.

## Data Availability

Data will be available on reasonable request.
